# Multimodal imaging of refractory *Candida* chorioretinitis progressing to endogenous endophthalmitis

**DOI:** 10.1186/s12348-015-0054-z

**Published:** 2015-08-08

**Authors:** Jeremy A Lavine, Mihai Mititelu

**Affiliations:** Department of Ophthalmology and Visual Sciences, University of Wisconsin-Madison, 2870 University Avenue, Room 206, Madison, WI 53705 USA

**Keywords:** Endophthalmitis, Chorioretinitis, *Candida albicans*, Optical coherence tomography, Fundus autofluorescence, Fluorescein angiography

## Abstract

**Background:**

Endogenous fungal endophthalmitis is a serious vision-threatening condition that occurs in immunosuppressed patients with candidemia.

**Findings:**

We report a complicated case of *Candida albicans* chorioretinitis that progressed to endophthalmitis. The patient required intravitreal and systemic anti-fungal medications with pars plana vitrectomy for successful treatment. Multimodal imaging using fundus photography, fluorescein angiography, spectral domain optical coherence tomography, and fundus autofluorescence was obtained throughout treatment. These modalities localized the *Candida* infection in the choroid, penetrating Bruch’s membrane, the retinal pigment epithelium, and the retina to enter the vitreous cavity. This infectious route resulted in loss of the retinal pigment epithelium, photoreceptors, and outer retinal layers, with scar formation that resulted in vision loss and increased future risk of choroidal neovascular membranes.

**Conclusions:**

Multimodal imaging of *C. albicans* chorioretinitis allows for accurate diagnosis, assessment of response to therapy, and prognosis for visual recovery and future complications.

## Findings

### Introduction

Endogenous fungal endophthalmitis is a serious vision-threatening condition. In the United States, yeasts are the most common causative organism, accounting for 75 % of cases [1]. Among yeasts, *Candida albicans* is the most common pathogen [1]. In 14 % of patients with Candidemia, ocular complications occur [2]. The majority of these patients develop chorioretinitis with 1.6 % of patients advancing to endophthalmitis [2]. In patients with yeast endophthalmitis, visual acuity outcomes can be poor with only 56 % of eyes achieving vision of 20/200 or better [1].

This case report describes the clinical course of a patient with *C. albicans* chorioretinitis that progressed to recalcitrant endophthalmitis. Using multimodal imaging, we chronicle the features of chorioretinitis through multiple medical and surgical therapies.

### Case Report

A 48-year-old male presented with blurred central vision in his right eye. Past ocular history included bilateral pseudophakia. Medical history was significant for a 4-year history of rheumatoid arthritis on oral prednisone 15 mg daily and a recent history of nephrolithiasis. One month prior to presentation, his nephrolithiasis was treated with extracorporeal shock wave lithotripsy and ureteral stent placement. One week after lithotripsy, urine cultures grew *C. albicans* and the patient started oral fluconazole 300 mg daily. For 3 weeks, the patient complained of blurred central vision in the right eye without redness or floaters.

**Fig. 1:**
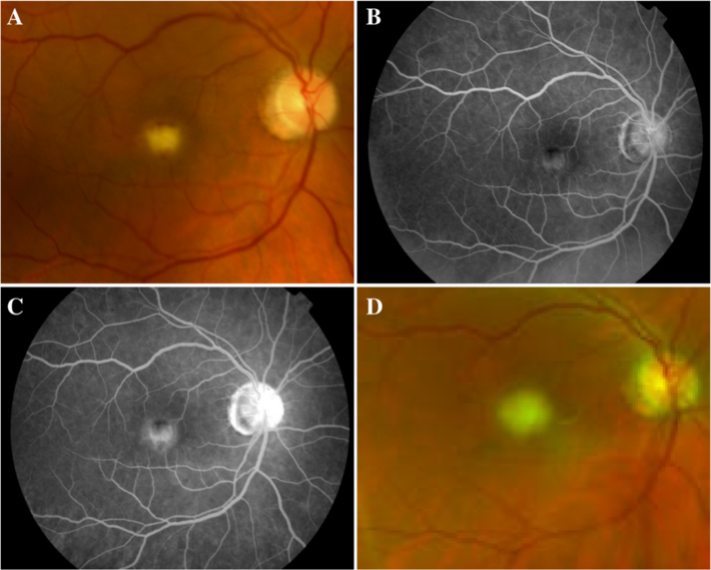
Initial appearance of the chorioretinitis. Color fundus photograph **(a)** showing the white, elevated, and fluffy chorioretinal lesion. Early **(b)** and late **(c)** phases of the FA demonstrating early hyperfluorescence and late staining. Color fundus photography 2 weeks later displaying significant vitritis that obscures retinal detail **(d)**

On examination, visual acuity was 20/100 OD and 20/20 OS. Pupils, intraocular pressures, and visual fields were normal. The anterior segment showed bilateral pseudophakia with no inflammation. Posterior segment exam demonstrated clear media without vitritis and a white, elevated foveal infiltrate (half disk diameter) with indiscrete borders (Fig. 1a). Fluorescein angiography (FA) displayed an early hyperfluorescent lesion with late staining (Fig. 1b, c). In the context of his Candiduria, a diagnosis of *Candida* chorioretinitis was made and his fluconazole was increased to 600 mg/day. The following day, the ureteral stent was removed without catheterization and blood cultures grew *C. albicans*. Although an infectious disease consult was requested on the day of presentation, the patient was seen 1 week later, and the fluconazole dose was increased to 800 mg daily.

The patient was lost to follow-up and presented 2 weeks later with new floaters OD. On examination, visual acuity remained 20/100 OD, the anterior segment was unchanged, and the posterior segment displayed vitritis with a fluffy chorioretinal lesion (Fig. 1d). *Candida* endophthalmitis was diagnosed, the patient received a same-day intravitreal injection of amphotericin B (5 μg/0.1 mL) and was placed on intravenous amphotericin B (0.1 mg/mL). The patient developed acute kidney injury (AKI) 1 week after initiation of treatment, requiring discontinuation of amphotericin B and resumption of fluconazole. At this time, blood cultures were negative for *C. albicans*.

**Fig. 2:**
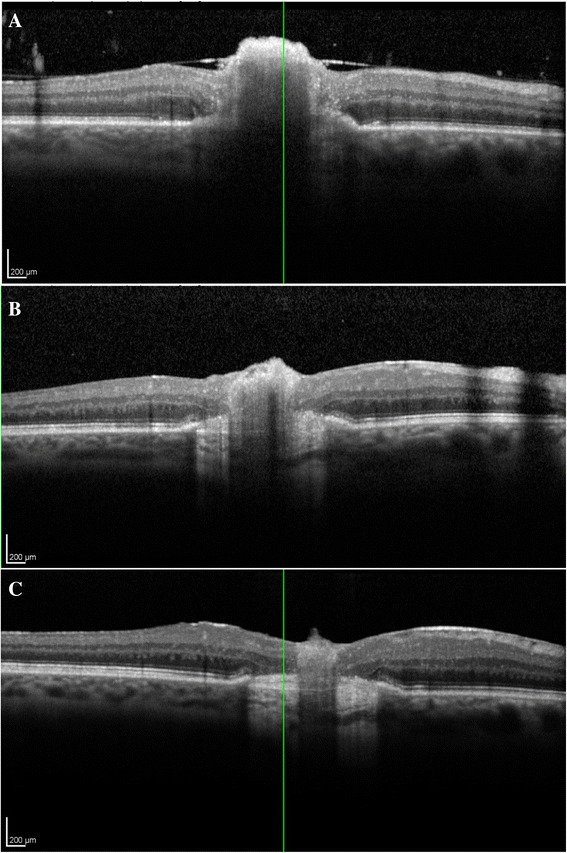
Regression of the chorioretinal lesion. SD-OCT imaging performed 1 week after intravitreal amphotericin B treatment **(a)**, 2 months after three intravitreal voriconazole injections **(b)**, and after PPV **(c)**. The inner retinal hyperreflective lesion regresses with time. At its borders, the RPE is elevated and discontinuous with loss of outer plexiform, outer nuclear, external limiting membrane, IS/OS, and RPE. Inner retinal tissues are preserved and subretinal scar is present

One week after intravitreal amphotericin B, there was improvement in the fluffy appearance of the macular lesion. Spectral domain optical coherence tomography (SD-OCT) demonstrated an elevated, hyperreflective foveal lesion at the vitreoretinal inferface that appeared to be emanating from the retinal pigment epithelium (RPE), with obscuration of the retinal layers (Fig. 2a). The patient received three intravitreal voriconazole injections (100 μg/0.1 mL) and was placed on systemic voriconazole (200 mg twice daily) therapy. After 2 months, the lesion flattened and developed well-demarcated borders but vitritis persisted. SD-OCT displayed an improving but still elevated hyperreflective lesion now localized at the nerve fiber layer with persistent focal discontinuity of the RPE (Fig. 2b). At its borders, the inner retinal architecture showed improved definition, but the outer retinal layers displayed atrophy with increased transmission defect. Due to the persistent vitreous infiltrates, the patient underwent pars plana vitrectomy (PPV). The postsurgical SD-OCT showed continued regression of the elevated, hyperreflective lesion (Fig. 2c). The borders demonstrated preserved inner retinal layers with atrophy of the outer retina and choroidal hyperreflectivity from transmission defect. The patient continued systemic voriconazole for an additional month without further intravitreal therapy.

**Fig. 3:**
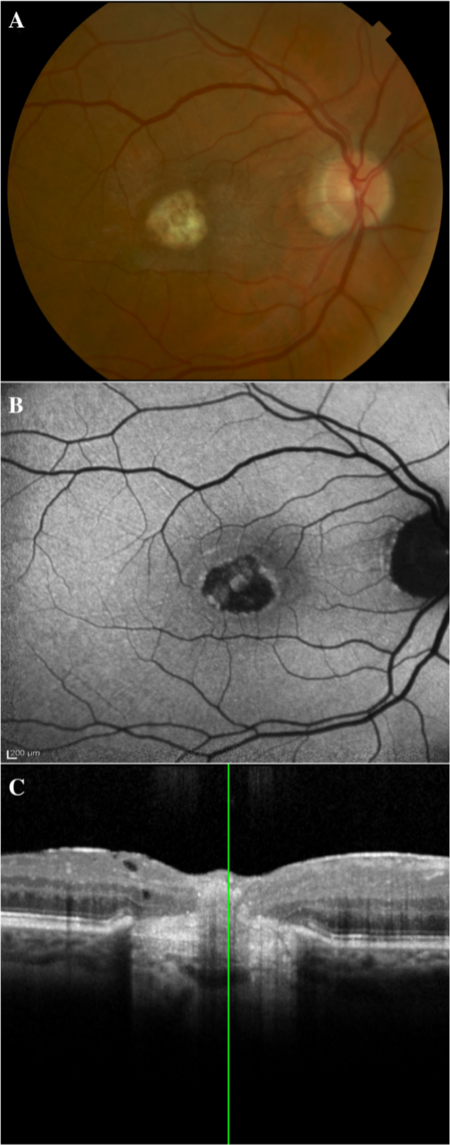
Final appearance of the chorioretinal lesion. Color fundus photography **(a)**, FAF **(b)**, and SD-OCT **(c)** were performed 2 months after PPV. A subfoveal scar is present **(a)** with destruction of outer plexiform, outer nuclear, external limiting membrane, IS/OS, and RPE **(c)**. The inner retinal layers, however, are preserved **(c)**. The FAF shows loss of autofluorsescence corresponding to RPE destruction **(b)**

Final multimodal imaging displayed a foveal scar (Fig. 3a). On fundus autofluorescence (FAF), the scar is hypofluorescent consistent with RPE loss (Fig. 3b). On SD-OCT, the outer retina is atrophic, the RPE layer is absent, a subretinal scar underlies the inner retinal layers, and a postinflammatory epiretinal membrane (ERM) co-exists (Fig. 3c). The photoreceptor loss and RPE atrophy primarily caused a final visual acuity of 20/100 OD.

### Discussion

We present a complicated case of *C. albicans* chorioretinitis that progressed to endogenous endophthalmitis, requiring multiple local and systemic antifungal medications and PPV. Risk factors for endogenous yeast endophthalmitis include hospitalization, surgery, cancer, diabetes, intravenous drug use, and indwelling catheters [1]. Risk factors for this particular case include recent lithotripsy of renal calculi and immunosuppression from prednisone therapy [3]. It is common for lithotripsy to cause asymptomatic Candiduria; in this immunocompromised patient, the Candiduria likely caused Candidemia, leading to chorioretinitis. In a prospective, multicenter study of 11 patients with chorioretinitis, none progressed to endophthalmitis [4]. Although limited by a small number of patients, this study suggests that our patient had been initially underdosed with fluconazole 300 mg daily. Once *Candida* endophthalmitis developed, our patient received both intravitreal and systemic amphotericin B. However, therapy was changed to oral and intravitreal voriconazole after AKI from amphotericin B. Voriconazole is effective against fluconazole-resistant *C. albicans* strains and has excellent ocular penetration [5]. It has been shown that *Candida* species preferentially infect the vitreous and form loculated microabscesses, which may ultimately require vitrectomy for clearance [6]. In the case of our patient, despite stabilization of the infection with aggressive systemic and intravitreal antifungal management, PPV was required to decrease the fungal load.

To our knowledge, this is the first report using FA, SD-OCT, and FAF to follow a case of *Candida* chorioretinitis progressing to endophthalmitis. Our patient initially presented with a white, elevated chorioretinal lesion (Fig. 1a). FA displayed a foveal lesion with early hyperfluorescence and late staining (Fig. 1b, c), consistent with prior reports of *Candida* chorioretinitis [7]. The lack of late leakage rules out choroidal neovascularization (CNV). However, the presence of vascular leakage near the lesion does not exclude *Candida* chorioretinitis as the cause of the macular lesion, as this angiogram pattern has been documented [8].

SD-OCT and FAF imaging help the clinician determine the route of infectious seeding, the etiology and treatment options, and the prognosis for visual recovery. SD-OCT findings early in the course of treatment demonstrated an elevated, hyperreflective lesion at the retina-vitreous interface with poorly defined borders and obscured underlying retinal detail (Fig. 2a). At the edge of the lesion, elevation of the RPE suggests that the lesion originated in the choroid. We suspect that this lesion is a focus of inflammatory and infectious material that locally infiltrated the macula. As the lesion was treated, the inner retinal hyperreflective lesion regressed but the borders demonstrated a persistently elevated, discontinuous RPE (Fig. 2b, c). These characteristics suggest that the *Candida* infection progressed via choroidal infiltration through Bruch’s membrane and RPE, into the retina and the vitreous. We hypothesize that this seeding is secondary to spread through the short posterior ciliary artery rather than through the central retinal artery [9,10] because of the initial presentation as an indolent chorioretinitis instead of an explosive endophthalmitis. Cho et al. previously evaluated *Candida* chorioretinitis with SD-OCT, demonstrating RPE elevation and outer retinal destruction in early, active lesions and inner retinal hyperreflective elevation with blockage in late, inactive lesions [11]. Our lesion showed SD-OCT characteristics of both early, active and late, inactive chorioretinitis stages as it evolved during the clinical course. We hypothesize that clinically and through multimodal imaging, our lesion demonstrated active features, especially given its pronounced hyperfluorescence on FA and its regression with treatment.

Vision loss in our patient occurred primarily due to photoreceptor loss and scarring. SD-OCT showed loss of outer plexiform, outer nuclear, external limiting membrane, and inner segment/outer segment (IS/OS) layers, and an area of thickened and hyperreflective subretinal scar tissue (Fig. 3c). FAF displayed lack of fluorescence centrally, indicating RPE destruction and confirming the presence of scar tissue (Fig. 3b). Not including the ring of hypofluorescent peripapillary atrophy, the scar is one disk diameter, which is twice the initial infiltrate. Thus, our imaging demonstrates centrifugal scar expansion and photoreceptor loss as the primary causes for vision loss. In other reports, vision loss occurred in the presence of macular edema [12,13], a clinical finding absent in this case.

Our patient remains at risk for further vision loss from potential CNV. FAF and SD-OCT imaging (Fig. 3) showed inflammatory and infectious destruction of Bruch’s membrane and RPE destruction, creating a locus for potential development of CNV. We suspect that once the integrity of the RPE layer has been violated, there is endophytic spread of the infection into the retina and vitreous, which increases the risk of further visual loss and the chance of requiring surgery. We emphasize that both SD-OCT and FAF highlight findings that were only previously demonstrable on histopathology. More importantly, they serve as non-invasive and readily available tools for monitoring progression and for informing the clinician regarding the effectiveness of various treatment modalities and prognosis for visual recovery.

## Abbreviations

AKI: acute kidney injury
CNV: choroidal neovascular membrane
ERM: epi-retinal membrane
FA: fluorescein angiography
FAF: fundus autofluorescence
IS/OS: inner segment/outer segment
OD: right eye
OS: left eye
OU: both eyes
PPV: pars plana vitrectomy
RPE: retinal pigment epithelium
SD-OCT: spectral domain optical coherence tomography
